# Supplementary material for: Implementation of an Enhanced Recovery after Surgery Protocol in Advanced and Recurrent Rectal Cancer Patients after beyond Total Mesorectal Excision Surgery: A Feasibility Study
Source: Cancers (Basel). 2023 Sep 12;15(18):4523. doi: 10.3390/cancers15184523 (PMC10526990; doi:10.3390/cancers15184523)
Supplement: Supplementary file 1 [file cancers-15-04523-s001.zip › cancers-2567214-supplementary.pdf]

SUPPLEMENTARY FILE – THE ERAS LARRC PROTOCOL IN DETAIL

The enhanced recovery programme for LARRC patients

After the evaluation of the current perioperative care and outcomes of patients with locally advanced and recurrent rectal cancer (LARRC), a standardised enhanced recovery programme (ERAS) for LARRC patients was developed. A literature study was performed to determine the perioperative aspects that could be improved in LARRC patients. Based on the information obtained in this literature study, the relevant aspects related to a standardised enhanced recovery programme in LARRC patients (e.g., drain management, urinary catheter management, multimodal pain management) were identified, discussed in multidisciplinary team meetings, and formed into a uniform ERAS protocol. These multidisciplinary team meetings consisted of at least a gastrointestinal surgeon, plastic surgeon, urologist, gynaecologist, anaesthesiologist, intensivist, physiotherapist, a general surgery nurse, oncology nurse, stoma care nurse and intensive care nurse. The resulting protocol was discussed with the colorectal cancer patient council for approval.

The ERAS LARRC Protocol

Preadmission

Patients should routinely receive standardised preoperative information, education, and counselling by the surgeon, anaesthesiologist, nurse practitioner, and stoma care nurse according to the colorectal ERAS protocol of Gustafson et al.[1] Preadmission optimisation, regarding intoxications, treatment of anaemia and nutrition, are added to this protocol according to the existing colorectal and pelvic ERAS protocol.[1,2]

Preoperative care

As "prehabilitation" in elderly patients is promising, elderly patients will receive physiotherapy treatment during 3 weeks before surgery to optimize their physical and nutritional status if their pre-existent condition was scored as frail using the PG-SGA©.[3–5]

Preoperative fasting was reduced to two hours before surgery for clear fluids, and six hours before surgery for solid foods.[6] Preoperative carbohydrates, up until two hours before surgery are supplemented to reduce the possibility of insulin resistance after surgery.[7,8] None of these patients will have a mechanical bowel preparation, only a rectal enema should be given, if deemed necessary, just before surgery.

The use of selective decontamination of digestive tract (SDD) in colorectal surgery remains controversial. However, it seemed to reduce the number of infectious complications.[9,10] As our retrospective cohort showed many infection-related complications (30.8%), the use of SDD was added as standard treatment to this protocol.

Intra-operative care

Anaesthesia protocol

An alternative anaesthesia protocol will be used, consisting of iv lidocaine (1.5mg/kg + 1.5mg/kg/h), iv methadone (0.3mg/kg) and iv ketamine (0.25mg/kg and 0.125mg/kg/h), and omitting the use of epidural analgesics. Postoperative pain will be managed by continuous wound infusion through wound catheters. These catheters are placed in the median laparotomy and the perineal wound.

Hyperglycaemia will be prevented by regular monitoring during surgery and corrected with insulin when needed (>10mmol/l).[11] Vasopressors, such as noradrenaline, will be used for correction of potential hypotension, and iv fluids should be used in maintenance volumes of 5-8ml/kg/h with the possibility to add 500ml hydroxyethyl starch in case of severe blood loss.

Postoperative nausea and vomiting (PONV) are to be prevented by the perioperative administration of dexamethasone 8mg iv and granisetron 2mg iv. Postoperative

administration of dexamethasone will be administered (daily 4mg iv) the first two days, and granisetron (3 times daily 2mg iv) during the first three days. Afterwards, in case of PONV, metoclopramide can be prescribed (3 times daily 10 mg oral) for a maximum of 5 days post-surgery.[12]

Laxatives, such as magnesium-oxide and macrogol should be administered standardly. Patients will receive chewing gum and are offered a cup of coffee to stimulate bowel movement.[13,14] In addition, patients should be encouraged to maintain their daily activities as much as possible by getting out of bed in the morning, brushing their teeth and starting the day sitting at a table and ending the day in a similar fashion, to prevent cognitive impairment and stimulate recovery, especially in elderly patients.[5]

Postoperative care

Postoperative pain management will be achieved using paracetamol and metamizole. Escape medication, such as sublingual fentanyl (100ug), or ketamine drip (5mg/h) should be available. Patients are allowed to receive short-acting opioids if needed when they are mobilising or experience more pain than usual. All the above will be monitored by a specialised "pain team", consisting of a specialised nurse and an anaesthesiologist.

Postoperative oral intake will be encouraged according to a standardised protocol, in which they receive 500ml of clear liquids on the day of surgery, 750ml of liquid, powdered or ground food on day +1, 1000ml on day +2, 1500ml on day +3 and after day 4 without limitations. This differs of the standard ERAS protocol intended for colorectal surgery, in which patients are offered solid food immediately postoperatively, as these patients frequently develop gastroparesis/paralytic ileus, and a gastric tube was replaced in 25 patients (33.3%) in 2019. In this protocol, one of the goals is to place no gastric tube postoperatively. Monitor potential nausea and vomiting intensively, so if patients show any signs of a gastroparesis/paralytic ileus, a gastric tube will still be placed if deemed necessary.

Patients are weighed daily to be able to adjust (iv) fluid therapy accurately. The first two postoperative days, glucose levels will be measured and corrected if needed.

Patients should not receive any intra-abdominal drains in general. In the very extended cases, or in case of a urologic reconstruction, an intra-abdominal drain is placed, which will be removed on postoperative day +2, when it produces less then 200ml per 24 hours and the substance is serous. If not, the operating surgeon is consulted, and the treatment plan will be adjusted.

For the prevention of deep venous thrombosis and pulmonary embolisms, patients receive low-molecular-weight heparin during the first 28 days after surgery, in combination with elastic compression stockings, as literature showed that 13.2% of the patients who underwent major abdominal or pelvic surgery, developed a venous thrombo-embolic event.[15,16]

As the innervation of the bladder is commonly damaged or partially resected in this type of surgery, patients experience urinary retention significantly more often after open rectal surgery then after colon surgery.[17] The use of suprapubic catheterisation, in case of expected retention, is safe, associated with less infection, re-catheterisation and discomfort than transurethral catherisation.[18,19] A standard transurethral catheter should only be placed in case of epidural anaesthesia and should be removed when the epidural is removed.

The mobilisation program consists of daily respiratory exercises, sitting on a chair for 5-15 minutes postoperatively on the day of surgery. On day +1, patients sit in a chair while eating (3x 15-30 minutes) and walk 100 meters. Day +2, they are eating in a chair (3x 15-30 minutes) and walk 100 meters 2-3 times a day. On day +3, they eat in a chair (3x 15-30 minutes), and are 2x 1 hour out of bed, and walk 3-4 times 100 meters. On day +4, they eat in a chair (3x 15-30 minutes), are 3 x 1 hour out of bed, and walk 4-5 times 100 meters.

Discharge and follow up

Patients are discharged from the hospital in case of functional recovery, determined as independently mobile at the preoperative level, sufficient pain control with oral medication alone, ability to maintain at least 50% daily required caloric intake, no intravenous fluid administration, no clinical signs of infection and a productive digestive system.

After 2 or 3 days, patients receive a call from the specialised colorectal surgical oncology nurses and after 30 days and 90 days after surgery, patients are seen in the outpatient clinic by the surgeon, the stoma care nurse and/or urologist on the same day.

SUPPLEMENTARY REFERENCES FOR THE PROTOCOL

1. Gustafsson UO, Scott MJ, Hubner M, Nygren J, Demartines N, Francis N, et al. Guidelines for Perioperative Care in Elective Colorectal Surgery: Enhanced Recovery After Surgery (ERAS(®)) Society Recommendations: 2018. World J Surg. 2019 Mar;43(3):659–95.
2. Nygren J, Thacker J, Carli F, Fearon KCH, Nordervall S, Lobo DN, et al. Guidelines for perioperative care in elective rectal/pelvic surgery: Enhanced Recovery After Surgery (ERAS(®)) Society recommendations. Vol. 37, World journal of surgery. United States; 2013. p. 285–305.
3. Han CY, Sharma Y, Yaxley A, Baldwin C, Miller M. Use of the Patient-Generated Subjective Global Assessment to Identify Pre-Frailty and Frailty in Hospitalized Older Adults. J Nutr Health Aging. 2021;25(10):1229–34.
4. van Rooijen S, Carli F, Dalton S, Thomas G, Bojesen R, Le Guen M, et al. Multimodal prehabilitation in colorectal cancer patients to improve functional capacity and reduce postoperative complications: the first international randomized controlled trial for multimodal prehabilitation. BMC Cancer. 2019 Jan;19(1):98.
5. Ghignone F, Hernandez P, Mahmoud NN, Ugolini G. Functional recovery in senior adults undergoing surgery for colorectal cancer: Assessment tools and strategies to preserve functional status. Eur J Surg Oncol J Eur Soc Surg Oncol Br Assoc Surg Oncol. 2020 Mar;46(3):387–93.
6. Brady M, Kinn S, Stuart P. Preoperative fasting for adults to prevent perioperative complications. Cochrane database Syst Rev. 2003;(4):CD004423.
7. Smith MD, McCall J, Plank L, Herbison GP, Soop M, Nygren J. Preoperative carbohydrate treatment for enhancing recovery after elective surgery. Cochrane database Syst Rev. 2014 Aug;(8):CD009161.
8. Carli F. Physiologic considerations of Enhanced Recovery After Surgery (ERAS) programs: implications of the stress response. Can J Anaesth. 2015 Feb;62(2):110–9.
9. Abis GSA, Stockmann HBAC, van Egmond M, Bonjer HJ, Vandenbroucke-Grauls CMJE, Oosterling SJ. Selective decontamination of the digestive tract in gastrointestinal surgery: useful in infection prevention? A systematic review. J Gastrointest Surg Off J Soc Surg Aliment Tract. 2013 Dec;17(12):2172–8.
10. Abis GSA, Stockmann HBAC, Bonjer HJ, van Veenendaal N, van Doorn-Schepens MLM, Budding AE, et al. Randomized clinical trial of selective decontamination of the digestive tract in elective colorectal cancer surgery (SELECT trial). Br J Surg. 2019 Mar;106(4):355–63.
11. Kwon S, Thompson R, Dellinger P, Yanez D, Farrohki E, Flum D. Importance of perioperative glycemic control in general surgery: a report from the Surgical Care and Outcomes Assessment Program. Ann Surg. 2013 Jan;257(1):8–14.
12. Weibel S, Rücker G, Eberhart LH, Pace NL, Hartl HM, Jordan OL, et al. Drugs for preventing postoperative nausea and vomiting in adults after general anaesthesia: a network meta-analysis. Cochrane database Syst Rev. 2020 Oct;10(10):CD012859.
13. Watanabe J, Miki A, Koizumi M, Kotani K, Sata N. Effect of Postoperative Coffee Consumption on Postoperative Ileus after Abdominal Surgery: An Updated Systematic Review and Meta-Analysis. Nutrients. 2021 Dec;13(12).
14. Short V, Herbert G, Perry R, Atkinson C, Ness AR, Penfold C, et al. Chewing gum for postoperative recovery of gastrointestinal function. Cochrane database Syst Rev. 2015 Feb;(2):CD006506.
15. Felder S, Rasmussen MS, King R, Sklow B, Kwaan M, Madoff R, et al. Prolonged thromboprophylaxis with low molecular weight heparin for abdominal or pelvic surgery. Cochrane database Syst Rev. 2019 Mar;3(3):CD004318.
16. Sachdeva A, Dalton M, Amaragiri S V, Lees T. Elastic compression stockings for prevention of deep vein thrombosis. Cochrane database Syst Rev. 2010 Jul;(7):CD001484.
17. Changchien CR, Yeh CY, Huang ST, Hsieh M-L, Chen J-S, Tang R. Postoperative urinary retention after primary colorectal cancer resection via laparotomy: a prospective study of 2,355 consecutive patients. Dis Colon Rectum. 2007 Oct;50(10):1688–96.
18. Buckley BS, Lapitan MCM. Drugs for treatment of urinary retention after surgery in adults. Cochrane database Syst Rev. 2010 Oct;(10):CD008023.
19. McPhail MJW, Abu-Hilal M, Johnson CD. A meta-analysis comparing suprapubic and transurethral catheterization for bladder drainage after abdominal surgery. Br J Surg. 2006 Sep;93(9):1038–44.
